# Quality Performance Indicators for the Surgical Management of Oesophageal Cancer: A Systematic Literature Review

**DOI:** 10.1007/s00268-023-07216-w

**Published:** 2023-10-22

**Authors:** Suheelan Kulasegaran, Yijiao Wang, Braden Woodhouse, Andrew MacCormick, Sanket Srinivasa, Jonathan Koea

**Affiliations:** 1 Norfolk And Norwich University Hospital, Colney Lane, Norwich, NR47UF UK; 2 Department of Surgery, North Shore Hospital, Auckland, New Zealand; 3 Department of Oncology, The University of Auckland, Auckland, New Zealand; 4 Department of Surgery, The University of Auckland, Auckland, New Zealand

## Abstract

**Background:**

The objective of this systematic review was to identify pre-existing quality performance indicators (QPIs) for the surgical management of oesophageal cancer (OC). These QPIs can be used to objectively measure and compare the performance of individual units and capture key elements of patient care to improve patient outcomes.

**Methods:**

A systematic literature search of PubMed, MEDLINE, Scopus and Embase was conducted. Articles reporting on the quality of healthcare in relation to oesophageal neoplasm or cancer and the surgical treatment of OC available until the 1st of March 2022 were included.

**Results:**

The final list of articles included retrospective reviews (*n* = 13), prospective reviews (*n* = 8), expert guidelines (*n* = 1) and consensus (*n* = 1). The final list of QPIs was categorized as process, outcome or structural measures. Process measures included multidisciplinary involvement, availability of multimodality diagnostic and treatment pathways and surgical metrics. Outcome measures included reoperation and readmission rates, the achievement of RO resection and length of hospital stay. Structural measures include multidisciplinary meetings.

**Conclusions:**

This systematic review summarizes QPIs for the surgical treatment of OC. The data will serve as an introduction to establishing a quality initiative project for OC resections.

## Introduction

Oesophageal cancer (OC) is a lethal condition with a 5-year population-based survival rate of less than 20% [1]. Significant variation in approaches to the surgical and multimodality treatment for patients with OC exists and may contribute to differences in patient outcomes. Quality performance indicators (QPIs) capture key elements of patient care that can be utilized to objectively measure the quality of care, identify underperforming providers across jurisdictions and develop benchmarking standards. This differs from published guidelines on the management of oesophagogastric cancers, which aim to guide clinicians and patients in making decisions about oesophagogastric cancer. QPIs should translate to improvements in short- and long-term clinical outcomes for patients. The optimal treatment pathway for patients with OC is complex and multidisciplinary [2]. Despite the availability of published guidelines for the management of OC, there is a relative paucity of well-defined evidence-based standards for the evaluation of the quality of surgical care [3].

There is strong evidence in support of the concentration of the surgical treatment of OC in high-volume centres [2, 4–6]. Nevertheless, it is important to decipher specific reasons for these improvements in the outcome as they serve as an impetus for ongoing quality improvement projects. Individual QPIs highlighted should ideally include patient-reported outcomes even though these might be difficult to define and measure.

The primary aim of this review is to identify and characterize existing QPIs for the surgical management of OC in the literature. These QPIs are imperative to capture performance across all aspects of patient care and outcomes. In addition to this, it will allow for the comparison of outcomes between different units and thus highlight underperforming units.

## Methods

Search terms included the following ‘Quality control OR Quality improvement OR Quality of healthcare OR Quality indicators’ OR ‘Benchmark’, AND ‘Oesophagectomy’ OR ‘Esophagectomy’ OR ‘Oesophageal neoplasm’ OR ‘Esophageal Neoplasm’ OR ‘Adenocarcinoma’ OR ‘Squamous Cell Cancer’ OR ‘Oesophageal resection’ OR ‘Esophageal Resection’ OR ‘Oesophageal cancer’ OR ‘Esophageal Cancer’ OR ‘Ivor Lewis’ OR ‘Oesophageal Surgery’ OR ‘Esophageal Surgery’. A systematic literature search of PubMed, MEDLINE, Scopus and Embase was conducted. All articles until the 1^st^ of March 2022 were included. The Preferred Reporting Items for Systematic Reviews and Meta-Analyses (PRISMA) statement was adhered [6]. Only papers in English were included, there were no geographical limits.

### Study selection and analysis

Articles that discussed, evaluated or reported on QPIs relating to the management of OC were included in the final analysis. Any study that solely reported surgeon or hospital case volume was excluded from the final analysis. Two authors independently reviewed the publications according to the inclusion criteria. Discrepancies were resolved by consultation. All QPIs were identified from the articles and sub-categorized according to the Donabedian model, which places QPIs into structural, process or outcome indicators. Structural indicators refer to the physical attributes of the healthcare setting. Process indicators measure the activity performed by the healthcare providers such as diagnostic and therapeutic interventions. Outcome indicators are the effects of the care [7].

Pertinent details including study size, methodology and QPIs were recorded and rated according to the Oxford Centre for Evidence–Based Medicine [8]. All studies were categorized based on the level of evidence.

*Level 1* Systematic reviews with homogeneity of randomized controlled trials. Individual randomized controlled trials.

*Level 2* Homogenous cohort studies or low-quality randomized controlled trials (e.g. with < 80% follow-up).

*Level 3* Systematic reviews with homogeneity of case–control studies or individual case–control studies.

*Level 4* Case series.

*Level 5* Expert opinion without critical appraisal.

## Results

**Fig. 1:**
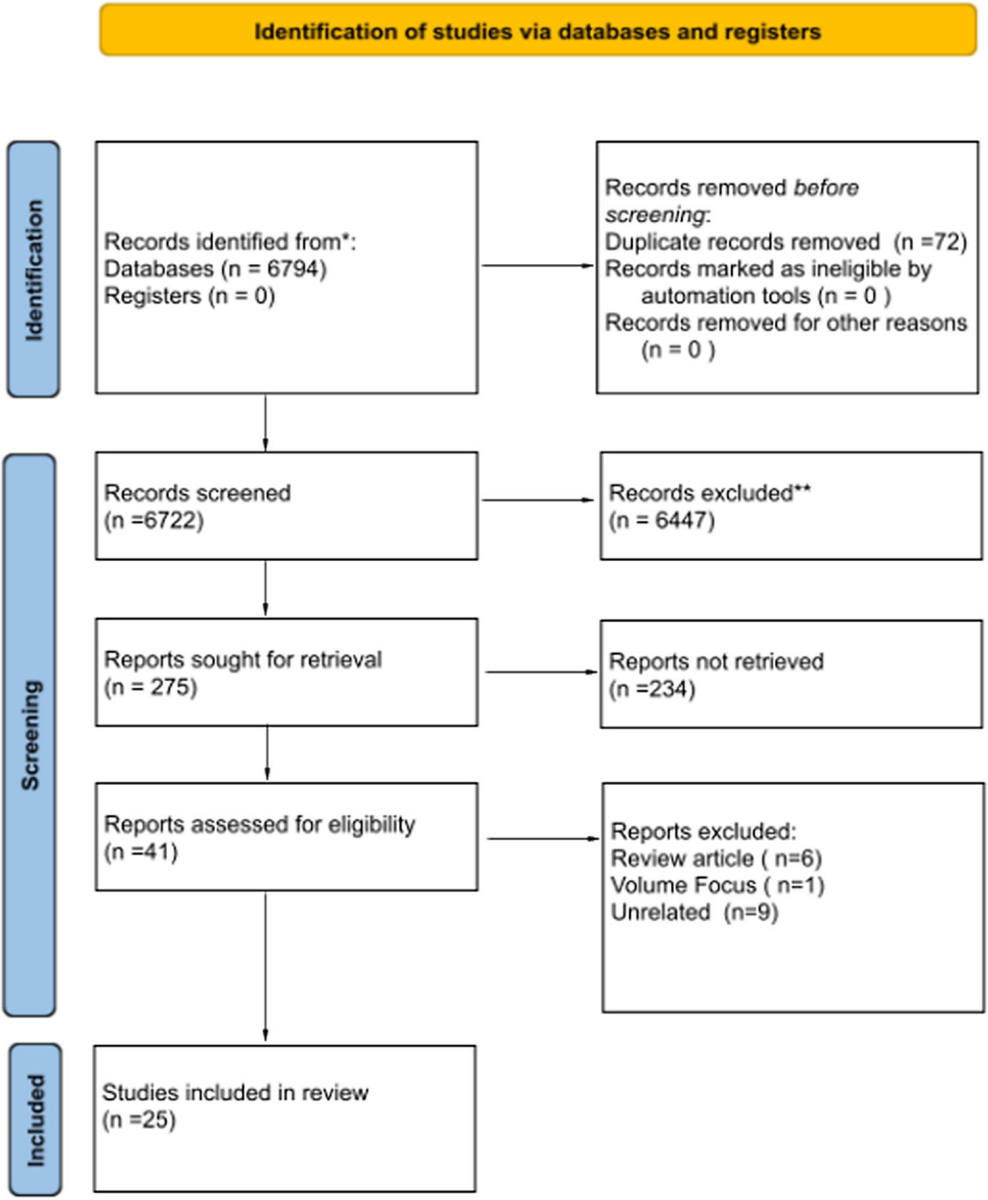
PRISMA [7] diagram describing the results of the systematic literature search and review

**Fig. 2:**
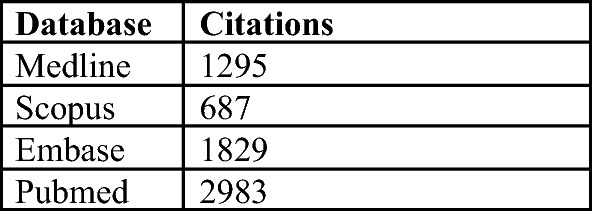
Hits per database detailing the yield for each database

A total of 6722 article titles were screened (Fig. 1). The search strategy is detailed in Fig. 2. The only structure-based QPIs reported were MDT and hospital patient/procedure volume [9]. Case volume was excluded from our analysis as per the pre-defined exclusion criteria. Twenty-three articles were included in the final analyses as shown in Table 1. Table 2 details the extracted process and outcome QPIs. The selected studies included retrospective review (*n* = 13), prospective review (*n* = 8), expert guidelines (*n* = 1) and consensus (*n* = 1).

**Table 1:** Summary of included publications describing QPIs and Oxford rating for each publication

Author	Year	Study design	Oxford rating	Site of research
Walters [21]	2014	Retrospective cohort Study	3	USA
Schlick [15]	2020	Retrospective review	3	USA
Adhia [9]	2020	Retrospective review	3	Europe
Samson [1]	2017	Retrospective review	3	USA
Burton [2]	2016	Prospective review	3	Australia
Staiger [22]	2018	Literature review	5	Switzerland
Low [37]	2019	Prospective review	3	Multinational contribution
Low [38]	2007	Prospective review	3	USA
Merkow [14]	2012	Retrospective review	3	USA
Carroll [18]	2020	Prospective review	3	Canada
Talsma [23]		Retrospective cohort study	3	Lebanon
Kalff [16]	2021	Consensus	5	Netherlands
Kulshrestha [27]	2020	Retrospective review	3	USA
Bolger [30]	2021	Retrospective review	3	Ireland
In [25]	2016	Retrospective review	3	USA
Schmidt [26]	2017	Prospective review	3	Switzerland
Busweiler [39]	Feb 2017	Retrospective review	3	Netherlands
Busweiler [19]	July 2017	Retrospective review	3	Netherlands
Valsangkar [40]	2018	Retrospective review	3	USA
Traverso [41]	2004	Prospective review	3	USA
Helminen [17]	2017	Prospective review	3	Finland
Markar [31]	2014	Prospective review	3	USA
Khoushal [42]	2016	Retrospective review	3	USA
Allum [43]	2018	Guidelines	5	Sweden

The site listed-USA; United States of America

**Table 2:** Publications reporting specific QPIs for the surgical management of oesophageal cancer

Type	Quality of care indicator	References
Structural	Multidisciplinary team care/conferences	[10]
Process	RO resection	[1, 9, 16, 18]
	Lymphadenectomy ≥ 15 nodes	[1, 9, 14, 15, 30, 39, 43]
	Administration of Induction Chemoradiation Therapy	[1, 2, 9, 27]
	Staging investigations CT neck/thorax/abdomen PET Staging laparoscopy	[2, 10, 43]
	Proportion of patients with OC beyond the mucosa (T2–4 NAny M0-1a) who received neoadjuvant treatment	[43]
	Proportion of patients diagnosed with cT1a OC undergoing endoscopic mucosal resection who had an en bloc resection	[43]
	Two or three-phase oesophagectomy	[43]
	Proportion of patients deceased with metastatic OC who received palliative support	[43]
	Accurate pathology reporting	[43]
	Proportion of patients diagnosed with recurrent OC discussed at the multidisciplinary team meeting prior to any treatment	[43]
	Pre-operative nutritional support	[43]
	Surgeon training	[41]
	Multidisciplinary esophagectomy care pathway	[11, 31]
Outcome	30-day mortality/90-day mortality	[2, 21, 24–26, 41]
	30-day/90-day comprehensive complication index	[26]
	In-hospital mortality	[16]
	Intraoperative blood loss	[41]
	Anastomotic leakage (all ECCG grades)	[16]
	No surgically related unplanned readmission within 30 days	[19]
	No reintervention	[30]
	No readmission related to surgical procedure	[16]
	Length of stay < 21 days	[30]
	No intraoperative complication	[30]
	Failure to rescue	[19]
	Pulmonary complications	[26]
	Severe post-operative complications (CD3 A and above)	[22]
	ICU readmission	[16]

Summary of reported and/or evaluated QPIs for surgical management of OC

### Structural indicator

#### Multidisciplinary team (MDT)

The delivery of high-quality OC management mandates multidisciplinary cooperation [9]. The efficient delivery and sequencing of different diagnostic and therapeutic interventions require discussion and documentation of all OC patients in an MDT meeting. Patient selection, investigation and management of patients with OC eligible for oesophagectomy by an MDT team resulted in improved survival compared to patients managed by surgeons alone [10]. The MDT should ideally include upper gastrointestinal surgeons, radiologists, pathologists and oncologists.

Formal standardized oesophagectomy care pathways and enhanced recovery pathways involving a multidisciplinary team including anaesthesia, intensive care, nursing, dietary and physical therapy was critical in achieving improved post-operative outcomes including median intensive care unit (ICU) stay, length of hospital stay and blood loss [11].

### Process indicators

#### Multimodality treatment

The role of induction therapy and the timing of surgery post-induction therapy for patients with OC are important quality indicators. Adhia et al. concluded that based on existing studies and guidelines, induction therapy in the form of chemotherapy and /or radiation to clinical stage III OC should be completed before surgery. In this group of patients, surgery should be performed no more than 60 days after the completion of induction therapy [9]. Samson et al. [1] corroborate the salient point that while early-stage patients may proceed directly to endoscopic or surgical resection, patients who are deemed operable with locally advanced (Stage IIb/IIIb) OC should be considered for induction therapy, typically chemoradiation followed by resection. Of note, patients receiving induction chemoradiation were less likely to have positive margins. This approach is supported by the National Comprehensive Cancer Network (NCCN) [12].

#### RO resection and lymphadenectomy

One of the quality indicators reflective of a successful oesophagectomy is the number of retrieved lymph nodes [13]. Most investigators emphasize the importance of adequate dissection, however, the exact number of lymph nodes required varied between studies. Most of the articles considered the evaluation of 15 or more lymph nodes ideal [1, 9, 14, 15].

However, some expert centres advocated for more lymph nodes to be sampled or to be sampled in a stage-dependent manner. For example, Kalff et al. [16] record 20 or more lymph nodes. Helminen used the benchmark value of 23 or more lymph nodes [17]. Achieving a complete surgical resection with negative microscopic surgical margins (R0 Resection) remains a key QPI for both early and locally advanced OC [1, 9, 18, 19].

#### Failure to rescue

Failure to rescue (FTR) is the failure or delay in recognizing and responding to a hospitalized patient experiencing complications from a disease process or medical intervention [20].

Busweiler et al. emphasized that ‘failure to rescue’ is the most important quality parameter after mortality and morbidity. In addition to this, the timely recognition and early effective management of complications have a great effect on post-operative mortality after a major surgical complication [19].

### Outcome indicators

Mortality is a key performance indicator in OC patients. Short- and long-term mortality rates are critical QPIs for patients undergoing oesophagectomy as deaths related to complications of surgery or cancer recurrence reflect the quality of surgical care delivered to the patient, and 30-day and 90-day mortality after oesophagectomy are well-documented performance indicators [18, 21–27]. Nevertheless, Talsma et al. [23] conclude that 90-day mortality rates are an improved quality indicator compared to the 30-day mortality rate and in-hospital mortality. Other outcome indicators were primarily focused on important post-operative complication rates such as an anastomotic leak, pulmonary complication rates, length of stay, readmission, reintervention rates as well as short- and long-term disease and overall survival rates.

## Discussion

Despite advancements in the management of OC, it remains a lethal malignancy with a relatively dismal prognosis. It is a significant global health issue with a 5-year survival rate of 20% overall [1] and less than 50% for locally advanced disease [28].

The modernization of surgical and endoscopic techniques for the management of early OC and complications have contributed to improved patient outcome. Nevertheless, much of the improvement in OC outcomes can be attributed to better patient selection through improved pre-operative staging and the delivery of multimodal induction therapy. The multidimensional nature of OC therapy has mandated the sequential coordination of care delivered by different specialist groups to ensure optimized outcomes. QPIs allow for the objective measurement of all aspects of the patient pathway.

Process QPIs were heterogenous but broadly fell into two groups. These were specifically accepted surgical metrics and the utilization of specialized staging, endoscopic and the active involvement of a multidisciplinary team. Process indicators included lymphadenectomy of 15 or more lymph nodes, the administration of induction therapy, the surgical approach including endoscopic and minimally invasive oesophagectomy and the utilization of specific oesophagectomy pathways [29]. The complexity and ever-evolving nature of modern diagnostic and therapeutic options for OC mandates the discussion of all OC cases at a specialized MDT. In addition to this, the early involvement of a palliative care team in an MDT is imperative albeit not commonly instituted. Although the added value can be hard to measure, it would seem intuitive as a significant proportion of patients with OC are non-operable and referred for the best supportive care [29].

Processes involving perioperative care including prompt identification and management of patients with surgical complications are significant QPIs. Failure to rescue patients with complications after OC surgery is an important QPI. The early identification and expedient management of complications is dependent upon several key factors including consultant-led services with clear escalation pathways, the availability of resources and expertise such as ICUs, theatre availability and diagnostic and interventional radiology services [19]. Other less measurable concepts that improve this include attitudes, behaviours and departmental culture. Early escalation and discussion with other senior surgeons combined with other specialities are key to ensuring early patient care.

Surprisingly, aside from the volume-outcome correlation and involvement of the MDT, validated structural QPIs for OC such as level of staffing, presence of a specific surgical ICU and staff skill composition were limited. The lack of QPIs in this area holds the promise of improvement. In addition to improved surgical technique and higher quality perioperative care, improvements in mortality and morbidity in high-volume units may be attributed to possessing standardized pathways as a central component of enhanced recovery programmes. Formal oesophagectomy care pathways have demonstrated promising results in improving perioperative care, post-operative mortality and operative textbook outcomes as well as improved efficiency in quality health care delivery. Textbook outcome measures encompass a bundle of clinical outcomes, which represents the ideal post-operative course in patients with OC [30]. In addition to this, care pathways have also been shown to reduce the length of stay and costs involved in oesophagectomy [31].

There is significant heterogeneity in the specific operative approach to a patient with resectable OC. The decision-making is complex and should consider tumour location, patient comorbidities, surgeon and institutional experience. Regarding operative approach and technique, a meta-analysis has shown no clear-cut difference in short- or long-term outcomes between different techniques [31]. Minimally invasive surgery for OC surgery improves patient recovery without jeopardizing the quality of oncological resection. The MIRO trial demonstrated a lower incidence of intraoperative and post-operative major complications, specifically pulmonary complications in the hybrid minimally invasive oesophagectomy group compared to open oesophagectomy without compromising oncological outcome [32]. This was supported by the TIME and RAMIE trial, which demonstrated no difference in disease-free and overall, 3-year survival [33, 34]. Despite its advantages, minimally invasive resection is not as widely disseminated as it is in colorectal surgery. This may be attributed to several factors including operative complexity, surgeon and lack of robust long-term data on patients undergoing minimally invasive oesophagectomy (MIO). There is limited evidence to suggest that MIO should be a QPI. Bolger et al. concluded that when MIO was included as an outcome measure, patients achieving a textbook outcome measure demonstrated improved overall survival rates. Appropriate surgeon training in ideally high-volume centres is critical in achieving high-quality surgery. A review by Stall et al. identified three studies reporting the influence of subspecialty training on outcomes. Two studies demonstrated lower post-operative mortality if operated on by cardiothoracic surgeons compared to patients operated on by general surgeons. A third study demonstrated no difference in outcome between general and thoracic surgeons. To date, there have been no studies comparing dedicated oesophagogastric surgeons and thoracic surgeons. This highlights the potential importance of surgical sub-specialization and areas of training [3].

Outcome measures included specific complication rates, post-operative morbidity, recovery and mortality at the 30-and 90-day post-operative mortality data. This is commensurate with the National Institute for Health and Care Excellence (NICE) guidelines, Dutch Upper GI Cancer audit group and Queensland Oesophagogastric Surgery Quality index including no intraoperative complications, tumour-negative resection margins, minimum 15 lymph nodes, no severe post-operative complications, no reintervention/readmission to ICU, no prolonged hospitalization (21 days or less) and no readmission after discharge or post-operative mortality [9, 35, 36].

One of the limitations of this study is that our search only identified healthcare-related QPIs and not patient-reported quality measures. This is increasingly being recognized as a cornerstone of OC surgical management. This is particularly important for the older, frail or palliative OC patient where the quality-of-life measures are imperative and must be incorporated into the greater framework of quality indicator measurements. Standard processes to prevent hospital-acquired complications such as deep venous thrombus and surgical wound infection rates are important considerations, which will be key QPIs for future studies.

The data from this review will be used to develop a set of internationally agreed and measurable QPIs for OC. QPIs that warrant further investigation include robotic approaches, ideal endoscopic therapeutic management of complications as well as the effect of further surgical subspecialty training. In conclusion, this paper summarizes the structural, process and outcome-based QPIs that are both clinically relevant and measurable. These QPIs can be utilized to provide objective measurements of outcomes and allow comparison between different units. These should ideally translate to improved short- and long-term patient outcomes and provide the basis for future quality improvement projects.

## References

[1] Samson P, Puri V, Broderick S (2017). Adhering to quality measures in esophagectomy is associated with improved survival in all stages of esophageal cancer. Ann Thorac Surg.

[2] Burton PR, Ooi GJ, Shaw K (2018). Assessing quality of care in oesophago-gastric cancer surgery in Australia. ANZ J Surg.

[3] Staal EFWC, Wouters MWJM, Boot H (2010). Quality-of-care indicators for oesophageal cancer surgery: a review. Eur J Surg Oncol.

[4] Birkmeyer JD, Stukel TA, Siewers AE (2003). Surgeon volume and operative mortality in the United States. N Engl J Med.

[5] Rouvelas I, Lagergren J (2010). The impact of volume on outcomes after oesophageal cancer surgery. ANZ J Surg.

[6] Gordon TA, Bowman HM, Bass EB (1999). Complex gastrointestinal surgery: impact of provider experience on clinical and economic outcomes. J Am Coll Surg.

[7] Rourke AJ (1957). Evaluating the quality of medical care. Hosp Prog.

[8] Centre for Evidence-Based Medicine (2023) OCEBM Levels of Evidence [Internet]. Nuffield department of primary care health sciences: university of Oxford; 2023 [Cited 5 June 2022.] Available from: https://www.cebm.net/2016/05/ocebm-levels-of-evidence/

[9] Adhia A, Feinglass J, Schlick CJ (2020). Adherence to quality measures improves survival in esophageal cancer in a retrospective cohort of the national cancer database from 2004 to 2016. J Thorac Dis.

[10] Stephens MR, Lewis WG, Brewster AE (2006). Multidisciplinary team management is associated with improved outcomes after surgery for esophageal cancer. Dis Esophagus.

[11] Markar SR, Karthikesalingam A, Low DE (2015). Enhanced recovery pathways lead to an improvement in postoperative outcomes following esophagectomy: systematic review and pooled analysis. Dis Esophagus.

[12] Ajani JA, D’Amico TA, Bentrem DJ (2019). Esophageal and esophagogastric junction cancer version 2. J Natl Compr Canc Netw.

[13] Matsuda S, Kataga Y (2021). The potential of lymph node yield as a quality indicator of esophagectomy for esophageal cancer. Ann Surg Oncol.

[14] Merkow RP, Bilimoria KY, Chow WB (2012). Variation in lymph node examination after esophagectomy for cancer in the United States. Arch Surg.

[15] Schlick CJR, Khorfan R, Odell DD (2020). Adequate lymphadenectomy as a quality measure in esophageal cancer: Is there an association with treatment approach?. Ann Surg Oncol.

[16] Kalff MC, van Berge Henegouwen MI (2021). Textbook outcome for esophageal cancer surgery: An international consensus-based update of a quality measure. Dis Esophagus.

[17] Helminen O, Mrena J, Sihvo E (2020). Benchmark values for transthoracic esophagectomy are not set as the defined “best possible”–a validation study. Ann Thorac Surg.

[18] Carroll PA, Jacob N, Yeung JC (2020). Using benchmarking standards to evaluate transition to minimally invasive esophagectomy. Ann Thorac Surg.

[19] Busweiler LA, Henneman D, Dikken JL (2017). Failure-to-rescue in patients undergoing surgery for esophageal or gastric cancer. Eur J Surg Oncol.

[20] Hall KK, Lim A, Gale B (2020) Failure to rescue. In: Making healthcare safer III: a critical analysis of existing and emerging patient safety practices. Agency for Healthcare Research and Quality (US), Rockville, pp 102–117 32255576

[21] Walters DM, McMurry TL, Isbell JM (2014). Understanding mortality as a quality indicator after esophagectomy. Ann Thorac Surg.

[22] Staiger RD, Gutschow CA (2019). Benchmark analyses in minimally invasive esophagectomy–impact on surgical quality improvement. J Thorac Dis.

[23] Talsma AK, Lingsma HF, Steyerberg EW (2014). The 30-day versus in-hospital and 90-day mortality after esophagectomy as indicators for quality of care. Ann Surg.

[24] Al Azzawi M, Bolger J, Whooley J (2020). Textbook surgical outcomes in esophageal cancer: the influence of national key performance indicators. Dis Esophagus.

[25] In H, Palis BE, Merkow RP (2016). Doubling of 30-day mortality by 90-days after esophagectomy: a critical measure of outcomes for quality improvement. Ann Surg.

[26] Schmidt HM, Gisbertz S, Moons J (2017). Defining benchmarks for transthoracic esophagectomy. Ann Surg.

[27] Kulshrestha S, Bunn C, Patel PM (2020). Textbook oncologic outcome is associated with increased overall survival after esophagectomy. Surgery.

[28] Ng SP, Leong T (2021). Indications for definitive chemoradiotherapy for oesophageal cancer. Ann Esophagus.

[29] Stordeur S, Vlayen J, Vrijens F (2015). Quality indicators for oesophageal and gastric cancer: a population-based study in Belgium, 2004–2008. Eur J Cancer Care.

[30] Bolger JC, Al Azzawi M, Whooley J (2021). Surgery by a minimally invasive approach is associated with improved textbook outcomes in oesophageal and gastric cancer. Eur J Surg Oncol.

[31] Markar SR, Schmidt H, Kunz S (2014). Evolution of standardized clinical pathways: refining multidisciplinary care and process to improve outcomes of the surgical treatment of esophageal cancer. J Gastrointest Surg.

[32] Mariette C, Markar SR, Dabakuyo-Yonli TS (2019). Hybrid minimally invasive esophagectomy for esophageal cancer. N Engl J Med.

[33] Straatman J, van der Wielen N, Cuesta MA (2017). Minimally invasive versus open esophageal resection: three-year follow-up of the previously reported randomized controlled trial: the TIME Trial. Ann Surg.

[34] Yang Y, Li B, Yi J (2022). Robot-assisted versus conventional minimally invasive esophagectomy for resectable esophageal squamous cell carcinoma: early results of a multicenter randomized controlled trial: the RAMIE trial. Ann Surg.

[35] Busweiler LAD, Wijnhoven BPL, van Berge Henegouwen MI (2016). The dutch upper GI cancer audit: 2011–2014. J Clin Oncol.

[36] Queensland Government (2017) Queensland oesophagogastric surgery quality index: indicators of safe, quality cancer care. Cancer surgery in public and private hospitals 2004–2013. Queensland Health, Brisbane

[37] Low DE, Kuppusamy MK, Alderson D (2019). Benchmarking complications associated with esophagectomy. Ann Surg.

[38] Low DE, Kunz S, Schembre D (2007). Esophagectomy- it’s not just about mortality anymore: standardized perioperative clinical pathways improve outcomes in patients with esophageal cancer. J Gastrointest Surg.

[39] Busweiler LA, Schouwenburg MG, van Berge Henegouwen MI (2017). Textbook outcome as a composite measure in oesophageogastric cancer surgery. Br J Surg.

[40] Valsangkar N, Salfity HB, Timsina L (2018). Operative time in esophagectomy: does it affect outcomes?. Surgery.

[41] Traverso LW, Shinchi H, Low DE (2004). Useful benchmarks to evaluate outcomes after esophagectomy and pancreaticoduodenectomy. Am J Surg.

[42] Khoushhal Z, Canner J, Schneider E (2016). Influence of specialty training and trainee involvement on perioperative outcomes of esophagectomy. Ann Thorac Surg.

[43] Allum W, Lordick F, Alsina M (2018). ECCO essential requirements for quality cancer care: oesophageal and gastric cancer. Crit Rev Oncol Hematol.

